# Special Issue “Latest Review Papers in Macromolecules 2025”

**DOI:** 10.3390/ijms262311740

**Published:** 2025-12-04

**Authors:** Xiao Hu, Giuseppe Zanotti, Salah-Eddine Stiriba

**Affiliations:** 1 Department of Physics and Astronomy, Rowan University, Glassboro, NJ 08028, USA; 2 Department of Biological & Biomedical Sciences, Rowan University, Glassboro, NJ 08028, USA; 3 Department of Biomedical Sciences, University of Padua, 35131 Padova, Italy; 4 Instituto de Ciencia Molecular/ICMol, Universidad de Valencia, *C*./Catedrático José Beltrán 2, 46980 Valencia, Spain

It is our pleasure to introduce this Special Issue (SI) of *International Journal of Molecular Sciences (IJMS)*, dedicated to highlighting contemporary developments and future perspectives in macromolecular science. The Macromolecules section of *IJMS* publishes both research articles and reviews that advance macromolecular studies in the fields of chemistry and biology. Macromolecular species are present everywhere in our lives, serving as the building blocks of all living organisms (i.e., proteins, carbohydrates, lipids, and nucleic acids) and forming the basis of synthetic macromolecule-based materials [1]. They perform vital functions in energy production, genetic information storage, and cellular structure formation, as well as being present in plastics, textiles, and other useful synthetic polymers [2]. The field of macromolecules is growing rapidly due to significant advancements in both data analysis and acquisition, as well as the development of new synthetic methods that allow greater control over their structure and function [3]. In this SI, seven review articles are included, collectively covering broad research domains including molecular imaging, biodegradable and bio-based polymers, enzymatic and protein biofunctionality, regulatory micropeptides, viral biomolecular interactions, and advanced 3D-printable biomaterials. We warmly invite readers to explore the full scope presented in this SI. These articles are introduced in chronological order as follows:

The first contribution, by Jia and coworkers (Contribution 1), overviews representative studies that utilize super-resolution microscopy (SRM) as a versatile tool for elucidating molecular assemblies, mainly focusing on the imaging of biomolecular assemblies made up of lipids, peptides, proteins, and DNA, as well as hybrid organic–inorganic and polymer assemblies. The review provides guidelines for the evaluation of the dynamics of molecular assemblies and assembly–disassembly processes with distinct dynamic behaviors through the application of SRM.

The review by Huang and coworkers (Contribution 2) provides an overview of recent progress and existing challenges in synthetic strategies, structural considerations, and mechanical and thermal properties of biodegradable polyester elastomers (BPEs) and their applications. Their potential as biodegradable elastomers to mitigate reliance on fossil resources, minimize waste generation, and decrease carbon emissions is highlighted. In fact, novel structures and properties of crosslinked BPEs can be achieved using bio-based ethanol and carboxylic acids.

Bechelany, Karav, and coworkers (Contribution 3) review the properties, functions, and potential applications of Lactoperoxidase (LPO), a member of the mammalian heme peroxidase superfamily and the first enzyme discovered in milk. They provide general information on this enzyme, including a comprehensive analysis that allows a better understanding of LPO’s biological significance with its potential applications.

The review by Shin, Min, and coworkers (Contribution 4) provides a comprehensive overview of the biogenesis of long noncoding RNA (lncRNA)-derived micropeptides, the methodologies for detecting and validating their expression, the molecular mechanisms governing their translation, and their emerging roles in metabolic regulation. By combining current findings and technological advancements on this topic, they highlight the potential physiological and pathological implications of these micropeptides and outline the future research directions in the field.

The properties, recent studies, and potential applications of Osteopontin (OPN), a known phosphorylated glycoprotein found in all vertebrate organisms and expressed in many tissues and secretions, were reviewed by Bechelany and Karav and their coworkers (Contribution 5) in this SI’s second review. They overview and compare the existing knowledge on the structure and functions of OPN with special emphasis on how milk-derived OPN impacts human and infants’ health and disease.

Wu and coworkers (Contribution 6) comprehensively explain the binding mechanisms between the capsid protein—which plays a crucial role in the viral life cycle—and the viral genome, as well as their potential applications as therapeutic targets. Special focus is given to small molecule inhibitors targeting capsid–genome binding interfaces.

This SI concludes with a review by Hu and coworkers on new frontiers in three-dimensional (3D) printing using biocompatible polymers (Contribution 7). They provide a comprehensive overview of the current state-of-the art 3D-printable biocompatible polymers and their composites, with emphasis on their processing methods, properties, and biomedical applications. The biocompatible polymers addressed in this review include both naturally occurring and artificial synthetic polymers, in addition to nanocomposite systems and bioinks.

The Macromolecules section of *IJMS* continues to provide an up-to-date platform for publishing innovative research and scientific advances. By presenting high-quality contributions from diverse scientific disciplines, we aim to broaden the understanding of macromolecular structure, function, and design. We invite researchers worldwide to continue sharing their cutting-edge findings and to join us in shaping the future landscape of macromolecular science.
